# Autochthonous tick-borne encephalitis virus (TBEV) - seropositive cattle in Belgium: a risk-based targeted serological survey

**DOI:** 10.1186/1756-3305-7-S1-O35

**Published:** 2014-04-01

**Authors:** S Roelandt, V Suin, F Riocreux, S Lamoral, S Van der Heyden, Y Van der Stede, B Lambrecht, B Cay, B Brochier, S Roels, S Van Gucht

**Affiliations:** 1 Unit for Coordination of Veterinary Diagnosis, Epidemiology and Risk Assessment (CVD-ERA), Operational Directorate of Interactions and Surveillance, Veterinary and Agrochemical Research Centre (VAR-CODA-CERVA), Brussels, Belgium; 2 National Reference Centre of TBEV and Rabies, Viral diseases, Communicable and Infectious Diseases, Scientific Institute of Public Health (WIV-ISP), Brussels, Belgium; 3 Unit for Data Management and Analysis (DMA), Operational Directorate of Interactions and Surveillance, Veterinary and Agrochemical Research Centre (VAR-CODA-CERVA), Brussels, Belgium; 4 Unit of Pathology and Prionology, Operational Directorate of Interactions and surveillance, Veterinary and Agrochemical Research Centre (CODA-CERVA), Brussels, Belgium; 5 Department of Immunology, Faculty of Veterinary Medicine, University of Ghent, Merelbeke, Belgium; 6 Unit of Avian Virology and Immunology, Operational Directorate of Virology, Veterinary and Agrochemical Research Centre (CODA-CERVA), Brussels, Belgium; 7 Unit of Enzootic and (Re-)emerging Diseases, Operational Directorate of Virology, Veterinary and Agrochemical Research Centre (CODA-CERVA), Brussels, Belgium

## 

Tick-borne encephalitis virus (TBEV) is the most important arthropod-borne virus in Europe. The Western subtype of this pathogenic neurotropic flavivirus is carried by *Ixodes ricinus*. Tick-borne encephalitis has become a considerable public health risk in several European countries, with currently 3000 hospitalized cases per year. The risk of TBEV-introduction into Belgium remains high and the presence of infected wildlife in Belgium is suspected. Domestic animals such as dogs or cattle can serve as excellent sentinels for TBEV-surveillance in order to install an early warning surveillance component for this emerging zoonotic disease of public health importance.

In a targeted, risk-based and cross-sectional sampling design, serological screening was performed on Belgian cattle (n = 650), selected from the 2010 Belgian national cattle surveillance serum bank. The three most Eastern provinces of Belgium, which are geographically situated closest to known and/or recently emerging TBEV-endemic, were targeted. These areas are also currently known as endemic for Lyme disease (*Borrelia burgdorferi*), another disease transmitted by the same tick.

Bovine sera were tested at the TBEV Belgian National Reference Centre at the WIV-ISP, by gold standard TBEV seroneutralisation test, based on the rapid fluorescent focus inhibition test (RFFIT) protocol. Using a conservative >1/15 cut-off titer for SN test, 17 bovines were seropositive and six had borderline results (1/10 < titer < 1/15). The accuracy of the RFFIT-SNT was confirmed in a mouse inoculation test and by West Nile virus and Rabies virus serology. There was a positive correlation between the neutralizing antibody titer, determined by SN, and the median survival time in mice inoculated intranasally with a mix of virus and serum. Lesions consistent of viral encephalitis were demonstrated in histopathology.

The overall bovine TBEV-seroprevalence in the targeted area was estimated between 2.6 and 4.3% and freedom could no longer be substantiated. Bovines with borderline results were often located close to confirmed seropositive animals. The geographical locations roughly coincided with the known Belgian hot spots for Lyme disease.

This risk-based cross-sectional serological survey in cattle, obtained through "one health" cooperation confirms the presence of infected foci in Belgium for the first time. Given the relevance of TBEV for the food chain through consumption of unpasteurized milk and cheese and through its considerable public health burden in other European countries, further surveillance in cattle, other sentinels, ticks and humans at risk is recommended to further determine the location and size of endemic foci and the risk for public health.

